# Metabolic engineering of *Clostridium autoethanogenum* for ethyl acetate production from CO

**DOI:** 10.1186/s12934-022-01964-5

**Published:** 2022-11-23

**Authors:** James C. Dykstra, Jelle van Oort, Ali Tafazoli Yazdi, Eric Vossen, Constantinos Patinios, John van der Oost, Diana Z. Sousa, Servé W. M. Kengen

**Affiliations:** Laboratory of Microbiology, Wageningen University and Research, Stippeneng 4, 6708 WE Wageningen, Netherlands

**Keywords:** Syngas fermentation, Ester, Ethyl acetate, Butyl acetate, Alcohol acetyl transferase, Acetogens

## Abstract

**Background:**

Ethyl acetate is a bulk chemical traditionally produced via energy intensive chemical esterification. Microbial production of this compound offers promise as a more sustainable alternative process. So far, efforts have focused on using sugar-based feedstocks for microbial ester production, but extension to one-carbon substrates, such as CO and CO_2_/H_2_, is desirable. Acetogens present a promising microbial platform for the production of ethyl esters from these one-carbon substrates.

**Results:**

We engineered the acetogen *C. autoethanogenum* to produce ethyl acetate from CO by heterologous expression of an alcohol acetyltransferase (AAT), which catalyzes the formation of ethyl acetate from acetyl-CoA and ethanol. Two AATs, Eat1 from *Kluyveromyces marxianus* and Atf1 from *Saccharomyces cerevisiae*, were expressed in *C. autoethanogenum*. Strains expressing Atf1 produced up to 0.2 mM ethyl acetate. Ethyl acetate production was barely detectable (< 0.01 mM) for strains expressing Eat1. Supplementation of ethanol was investigated as potential boost for ethyl acetate production but resulted only in a 1.5-fold increase (0.3 mM ethyl acetate). Besides ethyl acetate, *C. autoethanogenum* expressing Atf1 could produce 4.5 mM of butyl acetate when 20 mM butanol was supplemented to the growth medium.

**Conclusions:**

This work offers for the first time a proof-of-principle that autotrophic short chain ester production from C1-carbon feedstocks is possible and offers leads on how this approach can be optimized in the future.

**Supplementary Information:**

The online version contains supplementary material available at 10.1186/s12934-022-01964-5.

## Background

Short-chain esters, such as ethyl acetate, are important bulk chemicals used as organic solvents for the production of paints, coatings and resins [1–3]. The global ethyl acetate market was valued at $4.6 billion in 2021 and forecasted to grow to $8.3 billion by 2028 [4]. Traditionally, esters are produced by Fischer-Speier esterification of an organic acid and an alcohol [5]. This process relies on equilibrium reactions and demands high temperature and acidic conditions [6]. On the other hand, microbial biosynthesis of ethyl acetate from renewable or waste feedstocks offers a promising sustainable alternative [7].

Certain microorganisms are naturally capable of producing ethyl acetate, of which yeasts are well studied examples [7]. For instance, production of ethyl acetate by *Kluyveromyces marxianus* has been shown at pilot-scale with product yields up to around 50% [8]. The biosynthesis pathway for ethyl acetate formation in yeast utilizes alcohol acetyltransferases (AATs), which catalyze the condensation of acetyl-CoA and ethanol to ethyl acetate [7]. Atf1 and Eat1 are the two AATs that have been described to play a major role in the natural ethyl acetate formation [7, 9, 10].

Both Atf1 and Eat1 have been harnessed for metabolic engineering of various microbes to produce esters from sugars. Expression of either the *Wickerhamomyces anomalus* (Wan) Eat1 or the *Kluyveromyces marxianus* (Kma) Eat1 in *Escherichia coli* BW25113 Δ*ackA*Δ*ldhA* (DE3) both enabled up to 50% of the theoretical maximum yield of ethyl acetate from glucose [11]. Furthermore, truncation of Eat1 by removal of the mitochondrial pre-sequences resulted in an improved ethyl acetate yield and titer, i.e., 72% (C-mol ethyl acetate/C-mol glucose) and 43 mM, respectively, for the Wan Eat1 [11]. By contrast, expression of Atf1 in *E. coli* seems to result in the production of low amounts of ethyl acetate. For example, 0.1 mM ethyl acetate was produced with supplementation of 100 mM ethanol by *E. coli* TOPO expressing *Saccharomyces cerevisiae* (Sce) Atf1 grown in LB [12]. Furthermore, a yield of 5% was reported for ethyl acetate production from glucose by *E. coli* BW25113 Δ*ackA*Δ*ldhA* (DE3) expressing Sce Atf1 [11]. On the other hand, use of the Sce Atf1 is associated with high-level production of other short-chain esters such as butyl acetate [13]. For example, up to ca. 175 mM butyl acetate was produced from glucose by an engineered *Clostridium saccharoperbutylacetonicum*, representing 80% of the maximum theoretical yield [14]*.*

**Fig. 1:**
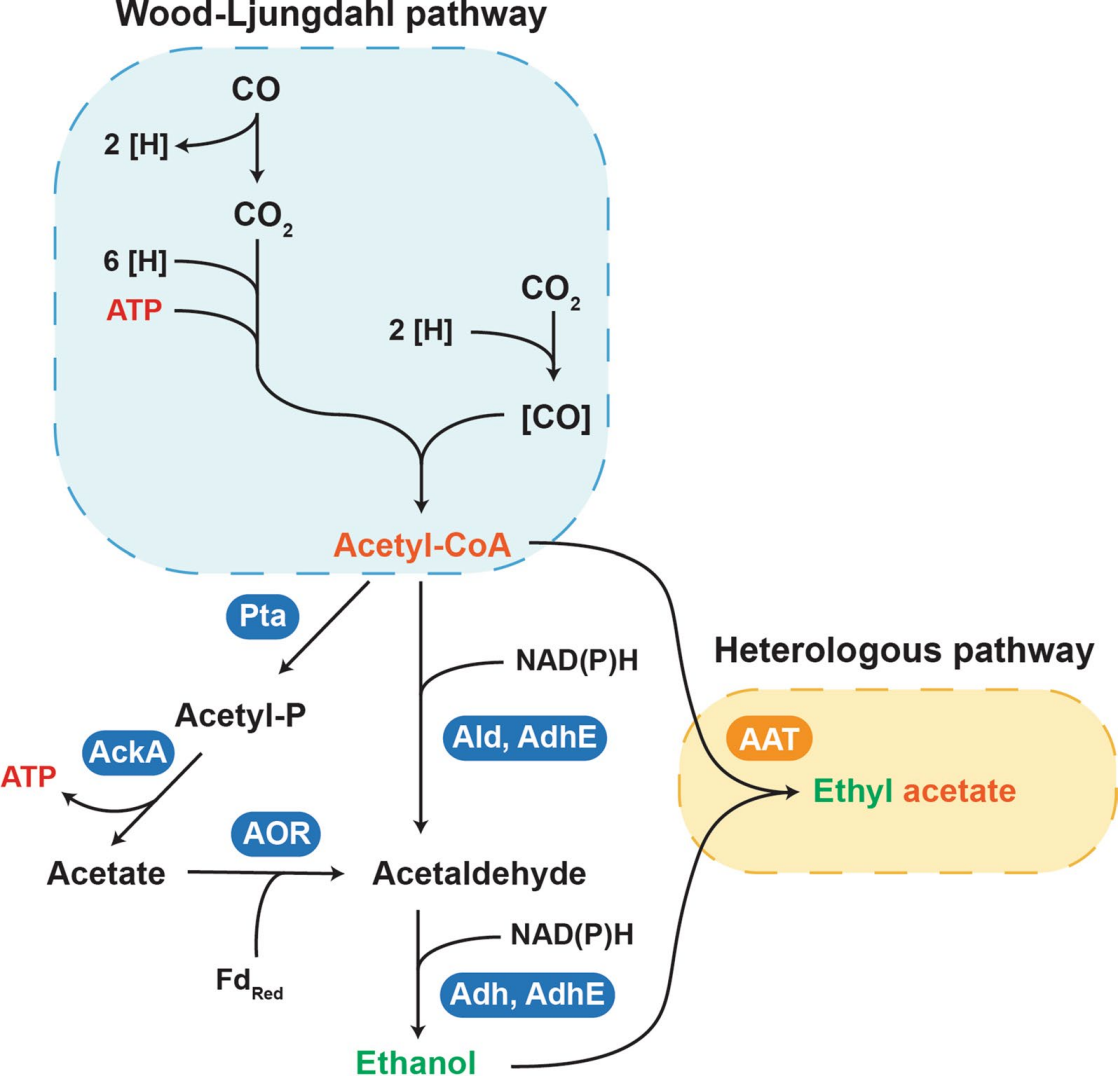
Central carbon metabolism of *C. autoethanogenum* and envisioned heterologous pathway for the production of ethyl acetate. Pta: phosphotransacetylase, AckA: acetate kinase, AOR: aldehyde:ferredoxin oxidoreductase, AdhE: bi-functional aldehyde/alcohol dehydrogenase, Ald: CoA-dependent acetaldehyde dehydrogenase, Adh: alcohol dehydrogenase and AAT: alcohol acetyltransferase

So far only sugar-based microbial production platforms have been explored for short-chain ester production. Utilization of more sustainable (waste) feedstocks and avoiding competition with food supply is therefore desirable. An alternative to sugar fermentation is gas fermentation, which utilizes gaseous carbon oxides (CO and/or CO_2_). These carbon oxides are abundantly available from industrial off-gases or from gasification of carbonaceous waste material (e.g., municipal solid waste) to syngas (CO, CO_2_ and H_2_) [15–17]. Anaerobic acetogens, such as *Clostridium autoethanogenum*, are amongst the best studied autotrophic bacteria for gas fermentation and make use of the Wood-Ljungdahl (or reductive acetyl-CoA) pathway for CO and CO_2_ (and H_2_) conversion [16, 18–21]. In *C. autoethanogenum*, CO and CO_2_/H_2_ conversion results in the formation of acetyl-CoA and subsequently ethanol; both precursors for a potential AAT-catalyzed ethyl acetate biosynthesis pathway (Fig. 1). Here, we report on the engineering of *C. autoethanogenum* for autotrophic ethyl acetate production from CO by expressing the AATs Kma Eat1 and Sce Atf1. By screening the two AATs in combination with two promoters and several production hosts, Atf1 was found to outperform Eat1 for ethyl acetate production. Additionally, we also established a proof of principle for production of other acetate esters from CO.

## Methods

### Bacterial strains and growth conditions

The bacterial strains used in this study are listed in Table 1. *E. coli* species were cultured aerobically in LB medium at 37 °C in the presence of the appropriate antibiotics (25 mg/L chloramphenicol with or without 50 mg/L kanamycin). Chemically competent *E. coli* DH5α (NEB, Ipswich, MA, United States) was used for general cloning, and electrocompetent *E. coli* sExpress (NEB Express carrying the conjugative plasmid R702) was used as donor for conjugation [22]*. C. autoethanogenum* JA1-1 (DSM 10061) was purchased from the Deutsche Sammlung von Mikroorganismen und Zellkulturen (DSMZ) GmbH (Braunschweig, Germany) and cultivated under strict anaerobic conditions.

**Table 1:** Bacterial strains and plasmids used in this study

Strain	Relevant characteristics	Source or reference
*E. coli* DH5α	F-endA1 glnV44 thi-1 recA1 relA1 gyrA96 deoR nupG Φ80dlacZΔM15 Δ(lacZYA-argF)U169, hsdR17(rK- mK +), λ–	NEB (New England Biolabs Inc., Ipswich, MA, United States)
*E. coli* sExpress	*fhuA2 [lon] ompT gal sulA11 R(mcr-73*:*miniTn10*–*TetS)2 [dcm] R(zgb-210*:*Tn10*–*TetS) endA1 Δ(mcrC-mrr)114*:IS10 carrying the Tra^+^, Mob^+^ plasmid R702 [R702-Tc^R^, Sm^R^, Su^R^, Hg^R^]	[22]
*C. autoethanogenum* JA1-1 (DSM 10,061)	Wild type	DSMZ
*C. autoethanogenum* ∆*pta*	∆*pta* (CAETHG_3358)	This study
*C. autoethanogenum* ∆*adhE1a*	Knockout of the Ald subunit of *adhE1* (CAETHG_3747)	This study
pMTL83151	pCB102, ColE1, *traJ*, Cm^R^/Tm^R^ (*catP*)	[23]
pMTL83122	pMTL83121 P_Thl_	[23]
P_Pta-AckA_-Atf1	pMTL83151 P_Pta-AckA_-*atf1*	This study
P_Pta-AckA_-Eat1	pMTL83151 P_Pta-AckA_-*eat1*	This study
P_Pta-AckA_-trEat1	pMTL83151 P_Pta-AckA_-tr*eat1*	This study
P_Thl_-Atf1	pMTL83122 P_Thl_-*atf1*	This study
P_Thl_-Eat1	pMTL83122 P_Thl_-*eat1*	This study
pRECas1	pMTL83151, P_fdx_*-RbE-*cas9*	[24]
pRECas1-*adhE1a*	pRECas1, sgRNA and HA targeting *adhE1a* (CAETHG_3747)	This study
pSIBR003	Int3-FnCas12a	[25]
pMTL-SIBR-Cas	pMTL83151, P_Fdx_- Int3-*FnCas12a*, P_AraE_-NTspacer	This study
pMTL-SIBR-Cas-*pta*	pMTL-SIBR-Cas with spacer and HA targeting *pta* (CAETH_3358)	This study

For all reported growth experiments, *C. autoethanogenum* was grown in modified ATCC medium 1754 PETC (ATCC, Manassas, VA, USA) at 37 °C. Modified ATCC medium 1754 PETC contained (per liter): 1.0 g NH_4_Cl, 0.1 g KCL, 0.2 g MgSO_4_, 0.8 g NaCl, 0.1 g KH_2_PO_4_, 0.02 g CaCl_2_ ⋅ 2H_2_O, 0.25 g sodium acetate, 1 g yeast extract, 20 g 2-(N-morpholino) ethanesulfonic acid (MES), 0.5 mg resazurin, 0.75 g L-cysteine ⋅ HCl, 40 mM fructose (only for heterotrophic growth), 10 mL trace element solution, and 10 mL Wolfe’s vitamin solution. The composition of the trace element solution (per liter) was 2 g nitrilotriacetic acid, 1 g MnSO_4_ ⋅ H_2_O, 0.8 g Fe(SO_4_)_2_(NH_4_)_2_ ⋅ 6H_2_O, 0.2 g CoCl_2_ ⋅ 6H_2_O, 0.2 mg ZnSO_4_ ⋅ 7H_2_O, 0.02 g CuCl_2_ ⋅ 2H_2_O, 0.02 g NiCl_2_ ⋅ 6H_2_O, 0.02 g NaMoO_4_ ⋅ 2H_2_O, 0.02 g Na_2_SeO_4_, 0.02 g Na_2_WO_4_ ⋅ 2H_2_O. The vitamin solution composition (per liter) was 2 mg biotin, 2 mg folic acid, 10 mg pyridoxine hydrochloride, 5 mg thiamine ⋅ HCl, 5 mg riboflavin, 5 mg nicotinic acid, 5 mg calcium pantothenate, 0.1 mg vitamin B12, 5 mg p-aminobenzoic acid, and 5 mg thioctic acid. Antibiotics were supplemented when required (6 mg/L or 15 mg/L thiamphenicol for autotrophic or heterotrophic growth, respectively).

For routine cultivation or growth on solid medium of *C. autoethanogenum* YTF medium was used that contained (per liter): 10 g yeast extract, 16 g tryptone, 10 g fructose, 0.2 g sodium chloride, 0.75 g L-cysteine ⋅ HCl, 0.5 mg resazurin, 10 mL ATCC medium 1754 trace element solution, 10 mL Wolfe’s vitamin solution, and 15 g agar (only for solid medium). When required, medium was supplemented with antibiotics (15 mg/L thiamphenicol and/or 250 mg/L D-cycloserine).

The growth experiments with *C. autoethanogenum* strains were carried out in 250 mL serum bottles containing 50 mL of modified ATCC medium 1754 PETC medium. The serum bottles contained either a gas phase of 170 kPa CO, or 40 mM fructose with a gas phase of 150 kPa N_2_. Strains were cultured in YTF before being transferred at approximately 5% (v/v) to precultures in ATCC 1754 PETC medium with CO or fructose as main carbon source. Precultures were transferred at least twice in ATCC 1754 PETC medium before used for growth experiments. For precultures with CO as carbon source, strains were incubated stationary until a decrease in total headspace pressure was measured, after which cultures were transferred to incubation at 150 rpm. Growth experiments were inoculated from these precultures at approximately 5% (v/v) and shaken at 150 rpm. For growth experiments involving supplementation of alcohols (ethanol, butanol, or hexanol) or butyl acetate, *C. autoethanogenum* strains were incubated one day until growth was observed prior to addition of the alcohols or butyl acetate. All growth experiments were carried out in biological triplicates. Sampling of cultures for analysis of final metabolite production was generally performed after depletion of substrate and/or growth arrest.

All mutagenesis work was performed in a Coy anaerobic chamber (Coy Laboratory Products, Inc., Ann.

Arbor, MI) with an atmosphere of approximately 1% H_2_ and 99% N_2_. Agar plates with *C. autoethanogenum* were incubated outside the anaerobic chamber at 37 °C in Mitsubishi™ AnaeroPack™ rectangular jars (Thermo Fisher, Waltham, MA) that contained an AnaeroGen™ sachet (Thermo Fisher). *C. autoethanogenum* freezer stocks were prepared by combining an equal volume of culture and 50% (v/v) glycerol in anaerobic vials reduced with a two to three drops of 100 mM titanium(III) citrate, and were stored at − 80 °C.

### DNA manipulations

Genomic DNA from *C. autoethanogenum* was isolated with the MasterPure™ Gram Positive DNA Purification Kit (Epicenter, Madison, WI) according to manufacturer’s protocol. PCR was carried out using either Q5® High-Fidelity DNA Polymerase (New England Biolabs, Ipswich, MA) for cloning purposes or OneTaq® DNA Polymerase (NEB) for diagnostic purposes according to the manufacturers’ protocols.

DNA fragments were isolated and purified from agarose gel using the Zymoclean™ Gel DNA Recovery Kit (Zymo Research Corporation) after gel electrophoresis or from PCR reaction mixture using the Thermo Scientific™ GeneJET PCR Purification Kit (Thermo Fisher), according to the manufacturer’s protocols. Plasmid DNA was isolated using the Thermo Scientific™ GeneJET Plasmid Miniprep Kit (Thermo Fisher, Waltham, MA) according to manufacturer’s protocol.

Plasmids were assembled either by using NEBuilder® HiFi DNA Assembly Master Mix (NEB), for which the protocol was modified by downscaling the total reaction mixture volume to 5 μL, or by using standard restriction ligation cloning techniques for which necessary restriction and ligation enzymes were purchased from NEB and used according to the manufacturer’s protocols.

All primers used in this study are listed in Table S1 (see Additional File 1). Primers were synthesized by IDT (Integrated DNA Technologies, Coralville, IA) or Sigma-Aldrich (Saint Louis, MO). Sanger sequencing of plasmids and amplicons was carried out by Macrogen (Macrogen Europe, Amsterdam, Netherlands).

### Plasmid construction

All plasmids used in this study are listed in Table 1. All plasmids are based on the *E. coli-Clostridium* shuttle vectors pMTL83151 or pMTL83122 [23] with pCB102, *catP*, *traJ* and *ColE*1 or *p15a*, respectively. The genes *eat1* (KMAR_10772, *Kluyveromyces marxianus* KM1777_03) and *atf1* (*Saccharomyces cerevisiae* CEN.PK113-7D) were codon harmonized with the algorithm described previously [26] for expression in *C. autoethanogenum* DSM 10061. The codon harmonized genes were synthesized by Twist Bioscience (San Francisco, CA). Sequences of codon harmonized genes *eat1* and *atf1* are available in Table S2 (see Additional File 1).

For construction of the AAT expression plasmids, the genes *eat1* and *atf1* and the truncated version of *eat1* were cloned into the multiple cloning site (MCS) of either pMTL83151 together with promoter P_Pta-AckA_ [27] or pMTL83122 downstream of P_Thl_. The aforementioned plasmids were linearized prior to cloning of the AAT genes via PCR amplification. The promoter P_Pta-ackA_ was amplified from *C. autoethanogenum* genomic DNA.

The vector pMTL-SIBR-Cas-*pta* for targeting of the *pta* (CAETHG_3358) gene was assembled as specified before [25] and described in more detail below. Plasmids maps for the plasmids used in this study can be found in the Additional File 2.

### Conjugation of C. autoethanogenum

Plasmids were transferred into the conjugative donor *E. coli* strain sExpress and subsequently conjugated into *C. autoethanogenum* according to previously established methods (Woods et al., [22]). Thiamphenicol (15 mg/L) was used to select for the *catP*-based plasmids. D-cycloserine (250 mg/L) was used to counter select against *E. coli* sExpress after conjugation.

### Generation of C. autoethanogenum knockout strains

The Ald domain of *adhE1* (CAETHG_3747), designated *adhE1a* as described before [28], was targeted using CRISPR Cas9/RiboCas gene editing techniques based on previously established methods [24]. The homology arms were composed of ca. 500 bp and 1 kb, upstream and downstream of the gene of interest, respectively. The synthetic guide sequence was designed using the software Benchling (https://www.benchling.com/crispr/). Assembly of the targeting vector pRECas1-*adhE1a* was achieved through traditional restriction ligation cloning methods of the sgRNA module and editing template, as described before [24].

The *pta* gene (CAETHG_3358) was targeted using the recently described SIBR-Cas gene editing technique [25]. The targeting vector was based on the backbone pMTL83151 with expression of the *FnCas12a* variant with Int3 [25] under control of the *fdx* promoter from *Clostridium sporogenes* [23], a crRNA under control of the *araE* promoter from *Clostridium acetobutylicum* [29] and homology arms of ca. 1 kb up- and downstream of *pta*. To insert the homology arms, the vector backbone was first digested with restriction enzymes KpnI and NcoI. The synthetic guide sequence for the crRNA was designed using the software Benchling.

The vectors pRECas-*adhE1a* and pMTL-SIBR-Cas-*pta* were sequence verified using Sanger sequencing before and after transformation to the conjugative *E. coli* donor strain sExpress, and subsequently conjugated into *C. autoethanogenum* as described previously [22]. Resultant colonies resistant to thiamphenicol and D-cycloserine were inoculated into liquid medium with thiamphenicol, and subsequently subcultured into liquid medium with thiamphenicol and 5 mM theophylline. After growth was observed, single colonies were isolated on solid media and were screened by PCR for the desired in-frame deletion in the *pta* or *adhE1* locus. The PCR product was sequenced to confirm the nature of the deletion (see Additional file 1, Figure S1, 2). Plasmid loss was achieved through serial passage in non-selective medium and confirmed through replica plating on medium with or without thiamphenicol as well as lack of amplification of the CRISPR-Cas plasmid in *C. autoethanogenum* knockout strain.

### Analytical techniques

Cell growth in liquid medium was monitored spectrophotometrically at 600 nm (OD_600_) using the spectrophotometer UV-1800 (Shimadzu, Tokyo, Japan). Headspace pressure was measured with a Greisinger GMH 3151 pressure reader (GSG Geologie-Service GmbH, Würzburg, Germany).

For analysis of metabolites, samples from the cultures were spun down by centrifugation (21,000 × g for 3 min, room temperature) after which the supernatant was stored at − 20 °C until analysis or analyzed immediately. Fructose, acetate, and ethanol were analyzed by HPLC with a LC-2030C Plus (Shimadzu) equipped with a Shodex SH1821 column operated at a temperature of 45 °C. The eluent used was 0.01 N H_2_SO_4_ at a flow rate of 1 ml/min. Peaks were detected with a refractive index detector. Relevant standards were used for identification and quantification. Ethyl acetate, butyl acetate, and hexyl acetate were analyzed by gas chromatography on a GC-2010 equipped with headspace sampler HS-20 (Shimadzu) and flame ionization detector. Supernatant (0.1 mL) from centrifuged culture samples (21,000 × g for 3 min, room temperature) was mixed with 0.1 mL internal standard (10 mM 1-propanol) in gas-tight glass vials. The vials were heated at 60 °C for 5 min and pressurized (100 kPa) with N_2_. The vial headspace was injected on an Agilent DB-WAX Ultra Inert column (30 m, 0.53 mm, 1.0 μm) with a split ration of 1 to 20. The column was operated at a constant pressure (30 kPa) with N_2_ as carrier gas and with a temperature gradient. The starting temperature was 70 °C which was held for 1 min followed by a temperature ramp of 50 °C/min to 125 °C and finally by a temperature ramp of 70 °C/min to 230 °C at which the temperature was kept until a total run time of 6 min was reached. Ethyl acetate was also measured by GC–MS with the GC Trace 1300 coupled with an EI Mass spectrometer ISQ 7000 and TriPlus RSH (Thermo Fisher). The GC–MS was equipped with an Agilent Porabond Q column (25 m, 0.25 mm, 3 μm) and operated with a flow of 1 ml/min of helium as carrier gas and a split of 50. Liquid samples of 0.2 mL were prepared in gas-tight glass vials, which were heated at 50 °C for 5 min after which 0.5 mL of the headspace was injected into the GC–MS. The column temperature was set to 100 °C for one min, followed by a ramp of 25 °C/min to 200 °C at which the temperature was kept for 3 min. Relevant standards were used for identification and quantification.

CO was analyzed by gas chromatography by injecting 0.2 mL of culture headspace into a CompactGC^4.0^ (Global Analyzer Solutions, The Netherlands). The CompactGC^4.0^ was equipped with a Carboxen 110 column (3 m, 0.32 mm) followed by a Molsieve 5A column, (30 m, 0.32 mm). The column oven temperature was set to 140 °C. The GC was operated with argon as carrier gas at a flowrate of 5 mL/min. A standard with a known amount of CO was used for identification and quantification of CO in culture samples.

## Results

### Heterologous expression of an AAT enables ethyl acetate production from CO

**Fig. 2:**
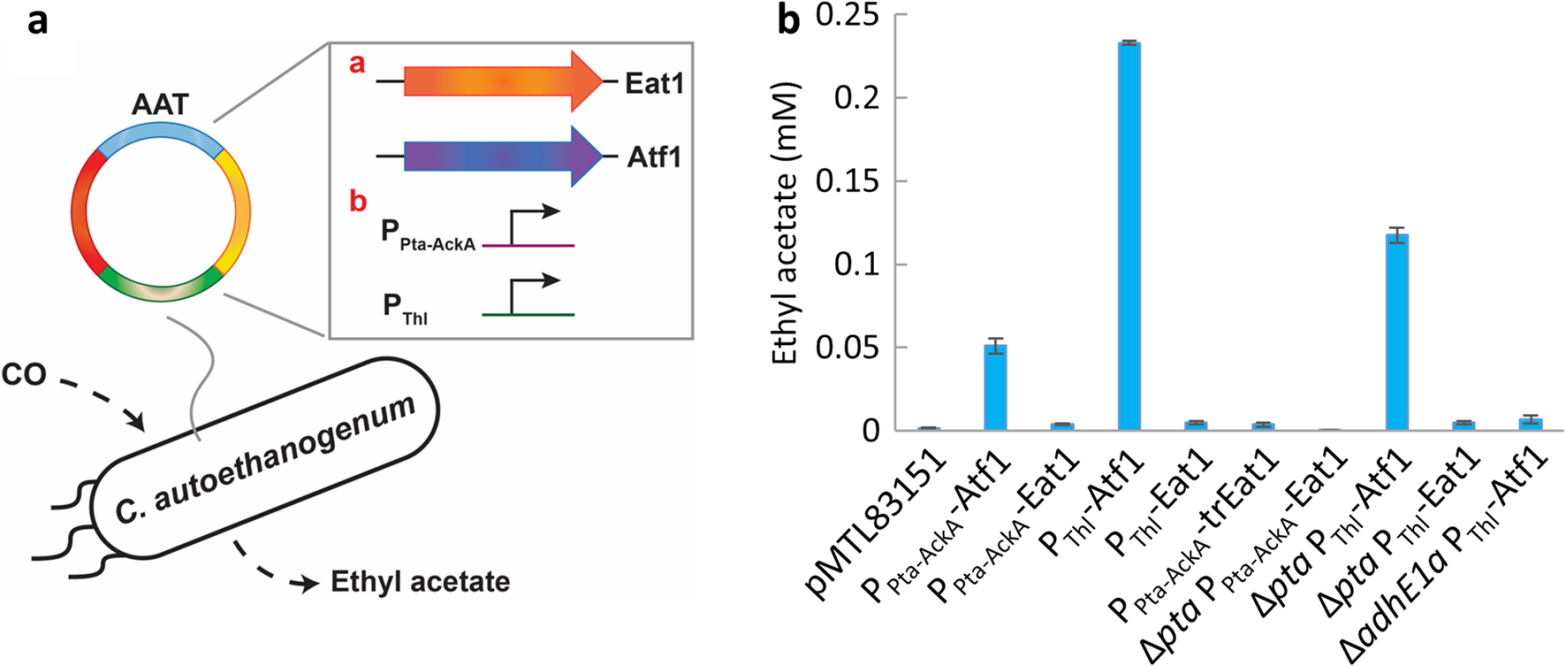
Screening of ethyl acetate production by *C. autoethanogenum* strains carrying plasmids for AAT expression. **A** Schematic illustration of engineered *C. autoethanogenum* strains with a: either Eat1 of Atf1 as AAT or b: either P_Pta-AckA_ or P_Thl_ as promoter. **B** Ethyl acetate production by the *C. autoethanogenum* strains grown on CO with pMTL83151 (empty plasmid control), P_Pta-AckA_-Atf1, P_Pta-AckA_-Eat1, P_Thl_-Atf1 or P_Thl_-Eat1. Data is represented as the average ± standard deviation of biological triplicates

To investigate the potential of producing ethyl acetate from CO by acetogenic bacteria, we initially screened different combinations of AATs, promoters, and expression hosts. The genes encoding the two AATs, Kma Eat1 and Sce Atf1, were each expressed from two different promoters: the *C. autoethanogenum* Pta-AckA promoter (P_Pta-AckA_) [27] and the *C. acetobutylicum* thiolase promoter (P_thl_) [23]. Besides the wild type Eat1, the truncated version of Eat1 [30] was included as well. The availability of the acetyl-CoA and ethanol precursors may also be important for the AAT-catalyzed formation of ethyl acetate. For instance, ethanol supplementation has shown to improve ethyl acetate production using Sce Atf1 [12]. Therefore, next to wild-type *C. autoethanogenum,* we also included *C. autoethanogenum* strains in which knockouts were made of either the phosphotransacetylase (*pta*) or the aldehyde dehydrogenase (Ald) subunit of the *adhE1* gene. Previously, disruption of the Ald subunit of *adhE1* has already been shown to enhance ethanol production from CO by nearly threefold [28]. Similarly, knockout of *pta* may also influence acetyl-CoA or ethanol metabolism (Fig. 1), and therefore ethyl acetate formation. The different *C. autoethanogenum* strains harboring the resulting plasmids for AAT expression or an empty plasmid control (pMT83151) were cultivated under autotrophic conditions with CO as main carbon source. The final ethyl acetate concentration was the highest for *C. autoethanogenum* [P_Thl_-Atf1] at approximately 0.2 mM ethyl acetate (Fig. 2). This corresponded to a final ethyl acetate yield of ca. 0.7% of the theoretical maximum on CO (see Additional file 1: Figure S3). Ethyl acetate formation was approximately fourfold lower for *C. autoethanogenum* [P_Pta-AckA_-Atf1] using the P_Pta-AckA_ rather than the P_Thl_ promoter for Atf1 expression. Only trace presence of ethyl acetate (< 0.01 mM) was detected for the *C. autoethanogenum* strains expressing Eat1, regardless of the promoter or Eat1 type (full length or truncated) used. No improvement in ethyl acetate production was observed for the Δ*pta* or Δ*adhE1a* strains, in fact, deletion of the Ald subunit of *adhE1* resulted in an unexpected decrease in ethyl acetate formation. Knockout of *pta* or *adhE1a* did not cause a substantial effect on growth or product spectrum under these conditions compared to wild-type *C. autoethanogenum* (data not shown). Screening of *C. autoethanogenum* strains harboring the Eat1 and Atf1 expression plasmids under heterotrophic conditions with 40 mM fructose as main carbon source showed a similar outcome to autotrophic growth; only Atf1-expressing strains produced ethyl acetate at low final concentrations (0.1 mM) (see Additional file 1: Figure S4). Furthermore, we also examined the potential detrimental ability of the production host to naturally degrade ethyl acetate esters by incubating ethyl acetate with and without *C. autoethanogenum*, but no ethyl acetate degradation was observed (see Additional file 1: Figure S5-6).

Next, we investigated the lack of ethyl acetate production by Eat1-expressing *C. autoethanogenum* strains by assaying these strains for Eat1 activity. Because measurement of Eat1 AAT activity in cell-free extracts has been proven to be difficult [10], Eat1 activity was assayed using in vivo alcoholysis, a secondary activity of Eat1 in which the alcohol moiety of an ester is replaced by another alcohol [31]. Together with exogenously supplied butyl acetate and endogenously produced ethanol, Eat1-catalyzed alcoholysis can be easily measured via ethyl acetate production. Varying concentrations of butyl acetate were added to cultures of *C. autoethanogenum* strains harboring plasmids P_Pta-AckA_-Eat1, P_Thl_-Eat1 or empty plasmid pMTL83151. However, neither of the Eat1-expressing *C. autoethanogenum* strains showed alcoholysis activity (see Additional file 1: Figure S7), suggesting that Eat1 may not be functionally expressed in *C. autoethanogenum.*

As another control, varying concentrations of butyl acetate were also added to cultures of *E. coli* strains harboring the same Eat1 expression plasmids as the *C. autoethanogenum* strains. In contrast to what was observed for *C. autoethanogenum*, *E. coli* strains showed Eat1-dependent alcoholysis activity in presence of butyl acetate (see Additional file 1: Figure S7). The Eat1-expressing *E. coli* strains also produced ethyl acetate in absence of butyl acetate, confirming Eat1-dependent AAT activity from the same plasmids used for the expression of Eat1 in *C. autoethanogenum*.

### Ethanol supplementation boosts ethyl acetate production

**Fig. 3:**
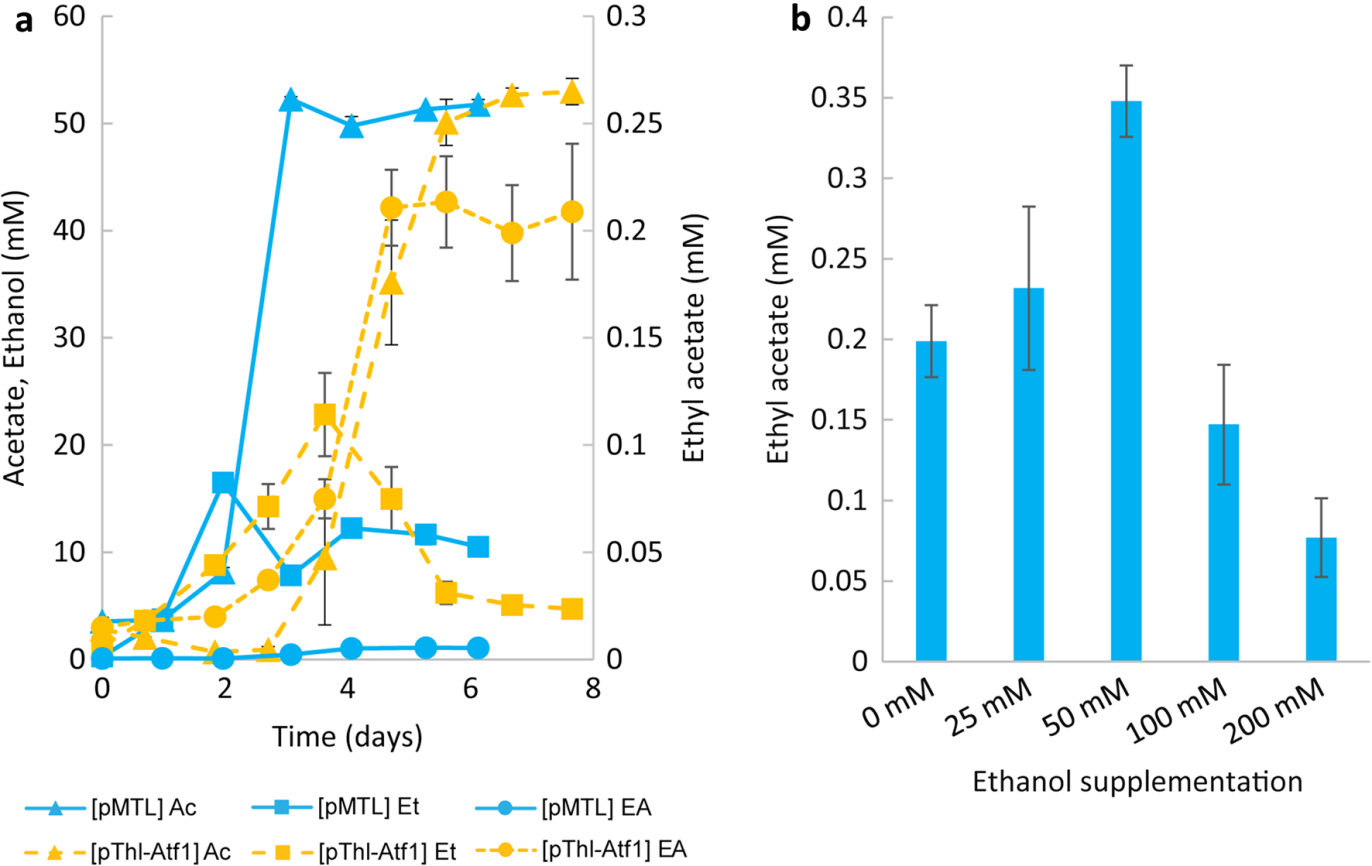
Investigating potential bottlenecks in ethyl acetate production in *C. autoethanogenum*. **A** characterization over time of *C. autoethanogenum* [pMTL83151] and [P_Thl_-Atf1] grown on CO. Ac: Acetate, Et: Ethanol, EA: Ethyl acetate. **B** Ethyl acetate production for *C. autoethanogenum* [P_Thl_-Atf1] incubated for 7 days on CO with ethanol supplementation at varying concentrations. Data is represented as the average ± standard deviation of biological triplicates

To gain further insights into autotrophic ethyl acetate production, potential metabolic bottlenecks in the currently engineered *C. autoethanogenum* strains were investigated. First, the best performing strain *C. autoethanogenum* [P_Thl_-Atf1] was characterized in more detail. For both *C. autoethanogenum* [pMTL83151] and *C. autoethanogenum* [P_Thl_-Atf1] the CO fermentation pattern was characterized by initial formation of ethanol, followed by acetate formation and ethanol consumption (Fig. 3a). Ethyl acetate did not show degradation over time. Because ethyl acetate production appeared to coincide with ethanol formation during part of the fermentation, ethanol was investigated as possible limiting precursor for ethyl acetate formation. Ethanol was supplemented to cultures of *C. autoethanogenum* [P_Thl_-Atf1] to final concentrations of 25 – 200 mM; these cultures were incubated with CO in the headspace for 7 days. Compared to the control (0 mM ethanol), cultures with 50 mM ethanol supplementation showed 1.5-fold increase in ethyl acetate production to approximately 0.35 mM (Fig. 3b). Higher ethanol concentrations were detrimental to growth and final ethyl acetate titers but appeared to enhance biomass-specific ethyl acetate production (see Additional file 1: Figure S8-9).

### Alcohol doping shows potential of Atf1 for ester production from CO

**Fig. 4:**
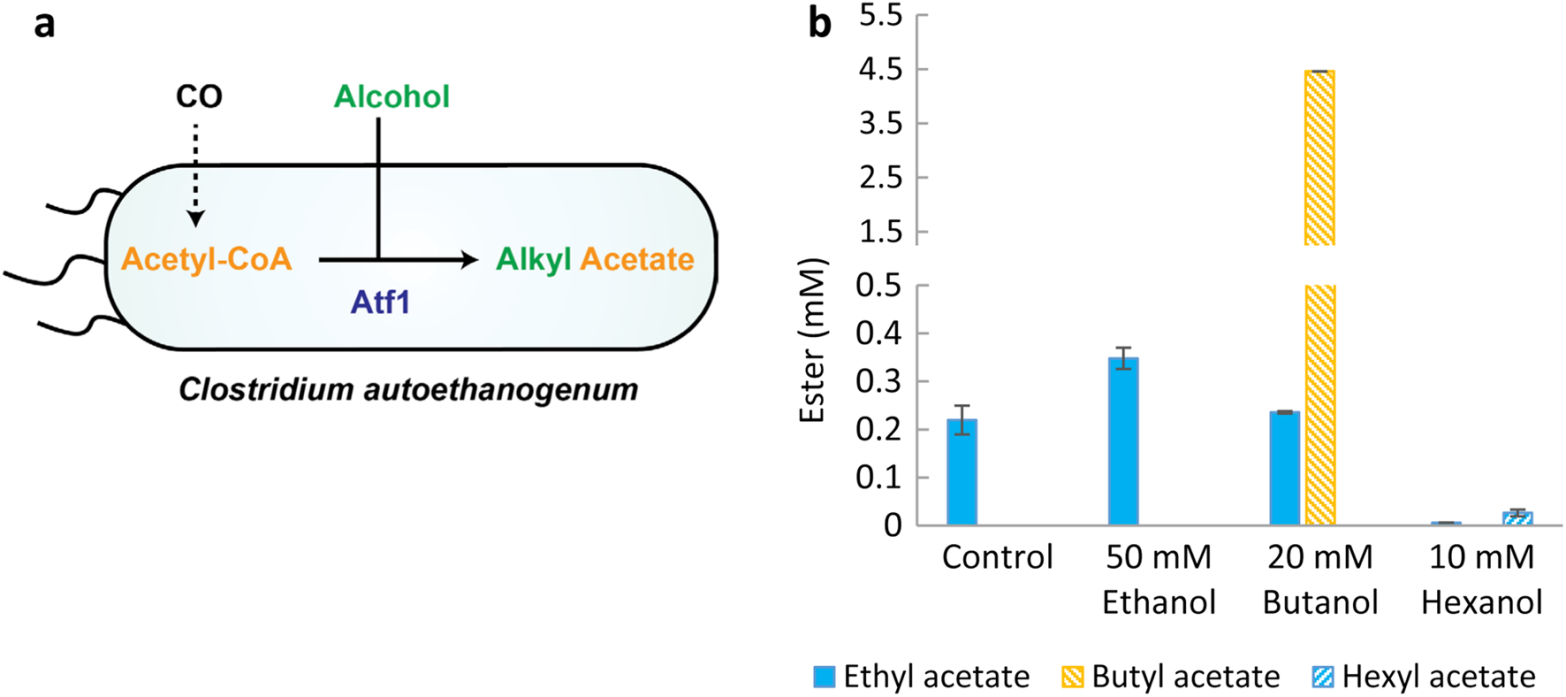
Acetate ester production from CO by C. autoethanogenum [P_Thl_-Atf1]. **A** Schematic representation of alcohol supplementation for acetate esters from CO. **B** Acetate ester production from CO with or without supplementation of ethanol, butanol or hexanol

Besides formation of ethyl acetate, Atf1 is known to have broad alcohol specificity and is able to catalyze formation of other acetate esters, including butyl acetate and hexyl acetate [13, 14, 32, 33]. To investigate whether an acetogen expressing Atf1 also has potential for biosynthesis of other acetate esters from CO, *C. autoethanogenum* [P_Thl_-Atf1] was grown on CO supplemented with either butanol or hexanol and was compared to a reference condition with and without ethanol supplementation (Fig. 4a). Strikingly, up to 4.5 mM of butyl acetate was formed in the presence of butanol. Only trace amounts (< 0.05 mM) of hexyl acetate could be detected in the presence of hexanol (Fig. 4b).

Compared to supplementation with ethanol, tenfold more total ester production was achieved with butanol supplementation. Although ethyl acetate production with and without the presence of butanol remained similar, 20-fold more butyl acetate than ethyl acetate was produced in the presence of butanol, representing a 95% selectivity for butyl acetate production.

## Discussion

We describe a successful proof-of-concept of engineering acetogens to produce ethyl acetate from CO as main carbon source. However, more efforts are needed before the envisioned autotrophic ethyl acetate production can compete with the conventional chemical catalysis or sugar-based microbial ethyl acetate production. Out of the two AATs tested, only expression of the Sce Atf1 resulted in ethyl acetate formation in *C. autoethanogenum*, regardless of the carbon source, background hosts, and promoters tested. The trace amounts of ethyl acetate observed for the empty plasmid control and Eat1-expression strains (but not for wild-type *C. autoethanogenum*) likely originated from the chloramphenicol acetyltransferase (CatP) encoded on the plasmids as antibiotic resistance marker [34]. The higher ethyl acetate production with the promoter P_Thl_ compared to P_Pta-AckA_ suggests that the P_Thl_ is stronger or more suitable for Atf1 expression on both CO and fructose. This may be specific to the microorganism and genes expressed. For instance, a study on autotrophic 2-propanol production from syngas in *Clostridium ljungdahlii* comparing the native *C. ljungdahlii* P_Pta-AckA_ and the P_Thl_ promoters showed that only the P_Pta-AckA_ enabled isopropanol production [35]. On the other hand, acetone production levels were similar comparing the P_Thl_ and the *C. ljungdahlii* P_Pta-AckA_ for pathway expression in *Acetobacterium woodii* grown on H_2_/CO_2_ [36].

Eat1 was a promising catalyst for ethyl acetate production based on previous work in *E. coli* [11]. However, Eat1 did not facilitate ethyl acetate production in *C. autoethanogenum* and was likely not functionally expressed based on lack of AAT and alcoholysis activity. Differences in AAT activity between Eat1 and Atf1 in *C. autoethanogenum* could be a result of differences in expression, enzyme characteristics or other factors. For instance, Atf1 was successfully expressed in *C. autoethanogenum* using the same promoters as for Eat1. This indicates that Eat1 might require other expression conditions than Atf1 for functional activity in *C. autoethanogenum*. Choice of inducible promoter and level of induction have previously been shown to strongly influence Eat1-catalyzed ethyl acetate production in *E. coli*, with Eat1 activity correlating positively with promoter strength [11]. Achieving functional expression of Eat1 in *C. autoethanogenum* might therefore benefit from a more extensive promoter library testing. Concerning enzyme kinetics, the affinity of purified Wan Eat1 for acetyl-CoA is relatively low (apparent *K*_*m*_ = 2.43 mM) compared to that of purified Sce Atf (apparent *K*_*m*_ = 45 μM) [10, 37]. This might indicate that Eat1 requires higher intracellular acetyl-CoA concentrations than Atf1. However, concentrations of intracellular metabolites, including acetyl-CoA, have been described to be similar between *E. coli* and *C. autoethanogenum* [38], and Eat1 can successfully catalyze ethyl acetate formation in *E. coli* [11]. Metabolic pathway optimization in *E. coli* lysates has also been shown to be representative for *C. autoethanogenum* [39, 40]. Yet there still may be physiological differences that affect activity of certain enzymes. For instance, the intracellular pH of the yeast mitochondria, where Eat1 is localized [41], is tightly regulated near pH 7.5 [42]. Atf1 appears to be located in the endoplasmic reticulum or cytosol [43–45], which have a pH near 7.2 [42]. Correspondingly, the intracellular pH of *E. coli* is near pH 7.6 [46]. However, the intracellular pH of clostridia like *C. autoethanogenum* and *Clostridium acetobutylicum* is significantly lower, near pH 6 [47–49]. Purified Sce Atf1 has a pH optimum near pH 7 but retains high activity at pH 6 [50]. For Eat1, the effect of pH on enzyme activity has not yet been investigated but it might be less suited for the acidic intracellular environment of clostridia. A previous study expressing the Wan Eat1 in another clostridial host, *Clostridium beijerinckii,* using the strong constitutive *C. beijerinkcii* P_thl_ also showed low AAT activity [31]. Curiously, *C. beijerinckii* expressing Wan Eat1 still showed high alcoholysis activity, in contrast to *C. autoethanogenum* expressing Kma Eat1 in this study. Host-specific functionality is further supported by the fact that Kma Eat1 was successfully expressed in *E. coli* using the same expression plasmids as for *C. autoethanogenum* in this study. However, host-specific expression elements, such as host-specific origins of replication, may contribute to this observation. Nevertheless, this also confirms previous findings that the promoters used are also active in *E. coli* [27, 51]. Overall, these results indicate that expression of Eat1 in Clostridia is troublesome and requires further investigation.

For Sce Atf1, only low final ethyl acetate titers were formed, despite ethanol supplementation, in line with previous observations [11, 12]. The inefficient ethyl acetate formation by Sce Atf1 might be due to low ethanol specificity, or other factors, such as suboptimal expression of Atf1. The first option is supported by our observations that, using the same Atf1 expression plasmid, nearly 20-fold more acetate esters could be produced with 20 mM butanol addition compared to a similar amount of ethanol addition. Therefore, this study indicates that the potential of Sce Atf1 may be limited for ethyl acetate production in acetogens under these conditions.

On the other hand, these experiments indicate that Atf1 might be an effective AAT to produce other acetate esters with acetogens from C1 substrates, such as butyl acetate or hexyl acetate. Surprisingly, the amount of butyl acetate produced in this study (4.5 mM) indicated that approximately 10% of the acetyl-CoA formed out of CO could be readily directed to this non-native product without further optimization. Hexyl acetate was only produced in trace amounts, comparable to earlier findings in *E. coli* expressing Sce Atf1 [32]. Besides butyl acetate and hexyl acetate, Atf1 is known to be able to catalyze formation of many other acetate esters from different alcohols such as isobutyl acetate or propyl acetate [32]. Research on acetogens has already focused on autotrophic production of a variety of alcohols such as butanol, hexanol, isobutanol, and isopropanol [39, 40, 52–59]. For efficient autotrophic production of non-native products using acetogens, the limited energy availability during autotrophic growth needs to be taken into account [60–63]. Importantly, production of acetate esters is ATP positive in acetogens on both CO and H_2_/CO_2_ assuming the bioenergetics for acetogens like *C. autoethanogenum* [49, 63]. Besides several alcohols, certain acetogens like *Clostridium carboxidivorans* can natively also produce longer-chain acyl-CoAs, such as butyryl-CoA, which expand the possibilities of autotrophic ester biosynthesis even further.

## Conclusions

We have shown that production of ethyl acetate from the gaseous C1 substrate CO can be successfully achieved by heterologous expression of an AAT in *C. autoethanogenum*. The highest ethyl acetate production was achieved using the Sce Atf1, which reached 0.2 mM ethyl acetate on CO, or approximately 0.7% of the theoretical yield. *C. autoethanogenum* expressing Atf1 was also able to produce other acetate esters from CO, including butyl acetate and hexyl acetate, when supplied with their respective alcohols. Although there is ample room for improvement, we provide a foundation for autotrophic production of acetate esters from one-carbon substrates.

## Supplementary Information

**Additional file 1: Table S1.** List of primers used in this study. **Table S2.** Sequences of genes expressed in this study. **Figure S1.** Image of Sanger sequencing results showed the nature of the in-frame deletion in the *pta* gene (CAETHG_3358). **Figure S2.** Image of Sanger sequencing results showed the nature of the in-frame deletion in the Ald subunit of the a*dhE1* gene (CAETHG_3747). **Figure S3.** Change in CO headspace pressure for *C. autoethanogenum* strains carrying plasmids for AAT expression on CO as main carbon source. **Figure S4.** Screening of ethyl acetate production by *C. autoethanogenum* strains carrying plasmids for AAT expression on 40 mM fructose. **Figure S4.** Investigating growth of *C. autoethanogenum* on CO and ethyl acetate. **Figure S5.** Investigating ethyl acetate degradation by *C. autoethanogenu*m grown on CO. **Figure S6.** Eat1 in vivo alcoholysis assay for *C. autoethanogenum* and *E. coli*. **Figure S7.** Investigating the effects of ethanol supplementation on growth of *C. autoethanogenum* [P_Thl_-Atf1] grown on CO. **Figure S8.** Investigating the effects of ethanol supplementation on ethyl acetate production by *C. autoethanogenum* [P_Thl_-Atf1] grown on CO

**Additional file 2**: zip. GenBank files of plasmids used in this study, including: ppta-acka-atf1.gb, ppta-acka-eat1.gb, ppta-acka-treat1.gb, precas1-ald.gb, pthl-atf1.gb, SIBR-Cas pta.gb.

## Data Availability

The datasets supporting the conclusions of this article are included within the article and its additional file.
