# Supplementary material for: Metabolic engineering of Clostridium autoethanogenum for ethyl acetate production from CO
Source: Microb Cell Fact. 2022 Nov 23;21:243. doi: 10.1186/s12934-022-01964-5 (PMC9686113; doi:10.1186/s12934-022-01964-5)
Supplement: Supplementary file 1 — Additional file 1: Table S1. List of primers used in this study. Table S2. Sequences of genes expressed in this study. Figure S1. Image of Sanger sequencing results showed the nature of the in-frame deletion in the pta gene (CAETHG_3358). Figure S2. Image of Sanger sequencing results showed the nature of the in-frame deletion in the Ald subunit of the adhE1 gene (CAETHG_3747). Figure S3. Change in CO headspace pressure for C. autoethanogenum strains carrying plasmids for AAT expression on CO as main carbon source. Figure S4. Screening of ethyl acetate production by C. autoethanogenum strains carrying plasmids for AAT expression on 40 mM fructose. Figure S4. Investigating growth of C. autoethanogenum on CO and ethyl acetate. Figure S5. Investigating ethyl acetate degradation by C. autoethanogenum grown on CO. Figure S6. Eat1 in vivo alcoholysis assay for C. autoethanogenum and E. coli. Figure S7. Investigating the effects of ethanol supplementation on growth of C. autoethanogenum [PThl-Atf1] grown on CO. Figure S8. Investigating the effects of ethanol supplementation on ethyl acetate production by C. autoethanogenum [PThl-Atf1] grown on CO [file 12934_2022_1964_MOESM1_ESM.docx]

# Supporting Information

Metabolic engineering of *Clostridium autoethanogenum* for ethyl acetate production from CO

**James C. Dykstra^1^, Jelle van Oort^1^, Ali Tafazoli Yazdi^1^, Eric Vossen^1^, Constantinos Patinios^1^, John van der Oost^1^, Diana Z. Sousa,^1^ and Servé W. M. Kengen^1^**

^1^Laboratory of Microbiology, Department of Agrotechnology and Food Sciences, Wageningen University and Research, Wageningen, Netherlands

Table S 1. List of primers used in this study.

| Primer | Sequence (5’ → 3’) | Additional information |
| --- | --- | --- |
| BG14051 | tgaatggcgaatggcgctag | pMTL80000 backbone |
| BG14052 | ccgcggtcatagctgtttcc |  |
| BG19579 | acgcgtccatggagatctc |  |
| BG17718 | catatgaactaacctcctaaattttgatacg | pMTL83122 backbone |
| BG14219 | ggaaacagctatgaccgcggCAATATGATATTTATGTCC | P_Pta-AckA_ |
| BG14220 | GTTCATTTCCTCCCTTTAAATTTAAC |  |
| BG21172 | GTTAAATTTAAAGGGAGGAAATGAACATGTTGCTAGCATATACAG | *eat1* with ol P_Pta-AckA_ |
| BG20028 | ctagcgccattcgccattcaTTAATCTCTTGCGCTTTTTAG |  |
| BG21195 | GTTAAATTTAAAGGGAGGAAATGAACatgAAAGGCTTGTTACCTTTACC | Tr*eat1* with ol P_Pta-AckA_ |
| BG21174 | GTTAAATTTAAAGGGAGGAAATGAACATGAATGAAATTGATGAGAAAAATC | *atf1* with ol P_Pta-AckA_ |
| BG20027 | ctagcgccattcgccattcaCTATGGACCTAACAATAGTG |  |
| BG19570 | aatttaggaggttagttcatATGAATGAAATTGATGAGAAAAATC | *atf1* with ol P_Thl_ |
| BG19571 | CGAGATCTCCATGGACGCGTCTATGGACCTAACAATAGTG |  |
| BG19575 | aatttaggaggttagttcatATGTTGCTAGCATATACAGTTAG | *eat1* with ol P_Thl_ |
| BG19578 | cgagatctccatggacgcgtTTAATCTCTTGCGCTTTTTAG |  |
| BG20860 | GGCCGTCGACcattaggctgcggttcctggGTTTTAGAGCTAGAAATAGCAAGTTAAAATAAGGCTAGTCCGTTATCAACTTGAAAAAGTGGCACCGAGTCGGTGCTTTTTTT | *ald* (CAETHG_3747) sgRNA |
| BG20858 | GGCCCCGGGACGTCATAAAAATAAGAAGCCTGCAAATGCAGGCTTCTTATTTTTATAAAAAAAGCACCGACTCGGTGCCACTTTTTCAAGTTG |  |
| BG21408 | tgcaagatttcaataaaggg | *ald* (CAETHG_3747) locus screening |
| BG21409 | ttaccttcattaatgacacc |  |
| BG20850 | GGCCGACGTCTCATCATCAgcataattatatcatgataacaattag | *ald* (CAETHG_3747) LHA |
| BG21081 | agcaaatttcttttgagcatc |  |
| BG21080 | gatgctcaaaagaaatttgcttctgtatccgaaaatgttgg | *ald* (CAETHG_3747) RHA |
| BG23938 | CCATGATTACGAATTCGAGCTCGGTACCtgatatgggagtaatattgct | *pta* (CAETHG_3358) LHA |
| BG23939 | CATGTTCATTTCCTCCCTT |  |
| BG23940 | AATTTAAAGGGAGGAAATGAACATGTAAtaacaaaaagcataaatg | *pta* (CAETHG_3358) RHA |
| BG23941 | CATGTCTGCAGGCCTCGAGATCTCCATGGaactgcaacataagctccta |  |
| BG24075 | aGGTCTCatagatcttttcctgctacttcacttccaAATTTaGAGACCa | *pta* (CAETHG_3358) spacer |
| BG24076 | tGGTCTCtAAATTtggaagtgaagtagcaggaaaagatctatGAGACCt |  |
| BG23943 | gccacatccagtagattgaac | *pta* (CAETHG_3358) locus screening |
| BG23942 | tgtgcttatttctagtgcctc |  |
| BG24803 | GGAGCAGAAATAGTAGATCCAGAG | *pta* (CAETHG_3358) screening internal |
| BG24804 | GCTTGAACTGCTGTTACAGCTACTAC |  |
| BG19777 | caggaaacagctatgaccgcggccgcTTAGTTATTCCTATTCTGCA | *cas12a* + Int3 |
| BG21633 | ATGTCCttgGGTTAATTGAG |  |
| BG21632 | CTCAATTAACCcaaGGACATTAACACACCTCCTTAAAAATTAC | *fdx* promoter |
| BG19860 | gtaccgagctcgaattcgtaatcatggtcatatgGTGTAGTAGCCTGTGAAATAAG |  |
| BG19781 | ggcccgcaccgatcgcccttcccaacagtttttatatttagtcccttgcc | *araE* promoter + spacer array (NT) |
| BG19782 | GAAATTGAGACCCTAGCGCGTATGGTCTCATCTACAACAGTAGAAATTaaaactcctccttaagatttatatatg |  |
| BG19783 | GAGACCATACGCGCTAGGGTCTCAATTTCTACTGTTGTAGATgcgcagcctgaatggcgaat |  |
| BG19784 | AACTGTTGGGAAGGGCGATCGG |  |

Table S 2. Sequences of genes expressed in this study

| ***Saccharomyces cerevisiae* CEN.PK113-7D Atf1 codon harmonized** |
| --- |
| ATGAATGAAATTGATGAGAAAAATCAGGCTCCCGTTCAACAAGAATGCTTGAAAGAGATGATACAGAATGGCCATGCACGAAGGATGGGTAGTGTAGAAGATTTGTATGTAGCACTAAATAGACAAAATTTATATCGTAATTTTTGCACATATGGTGAATTAAGCGATTATTGTACTAGGGATCAGCTAACATTAGCATTAAGGGAAATTTGCTTGAAAAATCCTACTTTGTTACATATAGTATTGCCTACAAGATGGCCTAATCATGAAAATTATTATCGTAGCAGCGAATATTATTCACGACCTCATCCTGTTCATGATTATATATCAGTTTTACAAGAATTAAAATTGAGCGGAGTTGTACTAAATGAACAACCAGAGTATAGCGCTGTTATGAAACAAATTTTAGAAGAATTTAAAAATAGCAAAGGAAGCTATACTGCTAAAATATTTAAATTGACTACAACTTTAACTATACCATATTTTGGTCCTACAGGTCCCAGCTGGCGATTGATATGTTTGCCTGAAGAGCACACAGAAAAATGGAAAAAATTTATTTTTGTTAGTAATCATTGCATGAGTGATGGACGAAGTAGCATTCACTTTTTTCATGATTTAAGAGACGAATTAAATAATATAAAAACTCCTCCTAAAAAATTAGATTATATATTTAAATATGAGGAGGATTATCAATTATTAAGGAAATTGCCTGAACCCATTGAAAAAGTTATTGACTTTAGACCTCCCTATTTATTTATACCCAAATCATTGTTGAGCGGATTTATTTATAATCATTTAAGATTTAGTTCAAAAGGAGTTTGTATGAGAATGGATGATGTTGAAAAAACAGATGATGTAGTTACAGAGATTATTAATATATCACCTACAGAATTTCAAGCCATAAAAGCTAATATAAAATCAAATATTCAAGGAAAATGTACTATTACTCCCTTTTTACATGTATGTTGGTTTGTTAGTTTGCATAAATGGGGAAAATTTTTTAAACCTTTAAATTTTGAATGGTTGACCGATATATTTATTCCCGCTGATTGCCGTTCACAATTGCCTGATGATGATGAAATGAGACAGATGTATAGATATGGTGCAAATGTAGGTTTTATAGACTTTACACCCTGGATTAGCGAATTTGACATGAATGATAATAAAGAAAATTTTTGGCCTTTGATAGAGCACTATCATGAAGTTATAAGCGAAGCATTAAGAAATAAAAAACATCTACATGGTTTAGGCTTTAATATTCAAGGTTTTGTACAAAAATATGTTAATATAGACAAAGTTATGTGCGATAGGGCTATTGGCAAAAGACGTGGTGGAACATTATTAAGCAATGTTGGATTGTTTAATCAGTTAGAGGAGCCCGATGCTAAATATAGTATTTGCGATTTAGCTTTTGGTCAATTTCAAGGTAGCTGGCACCAAGCTTTTAGCTTAGGAGTATGTAGCACTAATGTTAAAGGCATGAATATAGTAGTAGCATCAACAAAAAATGTAGTAGGAAGCCAAGAAAGTCTAGAAGAGTTGTGCAGCATATATAAAGCACTATTGTTAGGTCCATA |
| ***Kluyveromyces marxianus* KM1777_03 Eat1 (KMAR_10772) codon harmonized (lacking underlined nucleotides for truncation of Eat1)** |
| ATGTTGCTAGCATATACAGTTAGACCTAGTAATTGGAGTTTTACAAGAAGAGCTTATAGTGCAACTGCTAGAGCTTTTAATCAACAAAAAGGCTTGTTACCTTTACCTATTAAAGAAACTGTTGATATGGCTTATGATCTACATTTACCTGAAAGAAGTGTTATAGGAAAAATGCCTTATCATAGCCCTGAACCTATTATATTTTATCATGGATTATTGGGAAGTAAACGTAATTATAGACATGATTGTAAAAAATTGGCAACAGCATTACAAACTCCTGTTTATACTGTTGATATTAGAAATCATGGTAGTAGTGAACATGCATTGCCTTTTGATTATAATACTCTTGTTAATGATTTGGTTCATTTTGCTGAGACACATTCATTAGGAAAAGTAAATTTGGTTGGATATAGTTTAGGAGCAAAAGTAGCTATGTTGGCTTGTTTAAAACATCCTGAAAGGTTTAGCGCAGCATGTATAATAGATAATAGTCCTGAAGAACAACCACATATAAAACCATTATTGACTGCTTTGGTTAAAAGTTGCGTAAAATTATTGGATCATCATAATGTAAGAGCTGATGATAAATTGTGGAGACATAAAGCTAGCGAGGCTTTAAAAAAATATATACCTGATGCAGGAATTAGATATTATTTGCTTAGCAATATTATTAATAATCCAAGGGTTGTAGAATATAGAAGTCCTGTAATTAATTATGATGATGGAATGTTGCATTTTAAAAATCCTGTAAGACATATGATGGATTTTGTAACTAAAGAAGTAGCTGCATGGCCTACTCAGGAACTAGAGGGAAAACAATTTTTAGGTCCAGTAAATTTTATTAAAGCAACACGTAGTGATTTTATTAATCCTAAAAGCTTACAAGCTATAAATCAATATTTTCCATATCATAATATAGATGAAATAAATGCTACTCATTTTATTCTTAATGAAAGGCCACAAGAGTATCTTAGAGCTGTAACAGATTTTTTTAAAGTTACAAGATATCAACTTGAGAAAAAAAGAGAACAAGATTTGGCAAAAATTGATCAACTAAATGCTAGTGAAAGTCTAAAAAGCGCAAGAGATTAA |


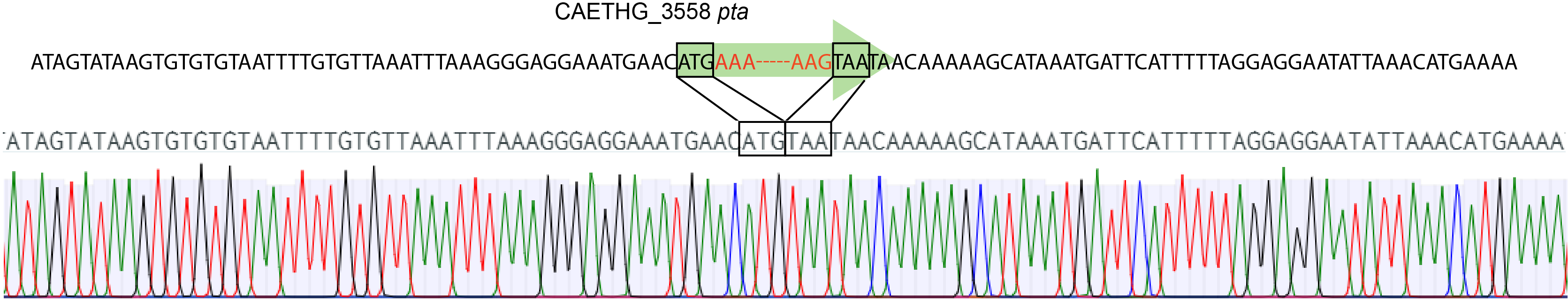


Figure S 1. **Image of Sanger sequencing results showed the nature of the in-frame deletion in the pta gene (CAETHG_3358).**


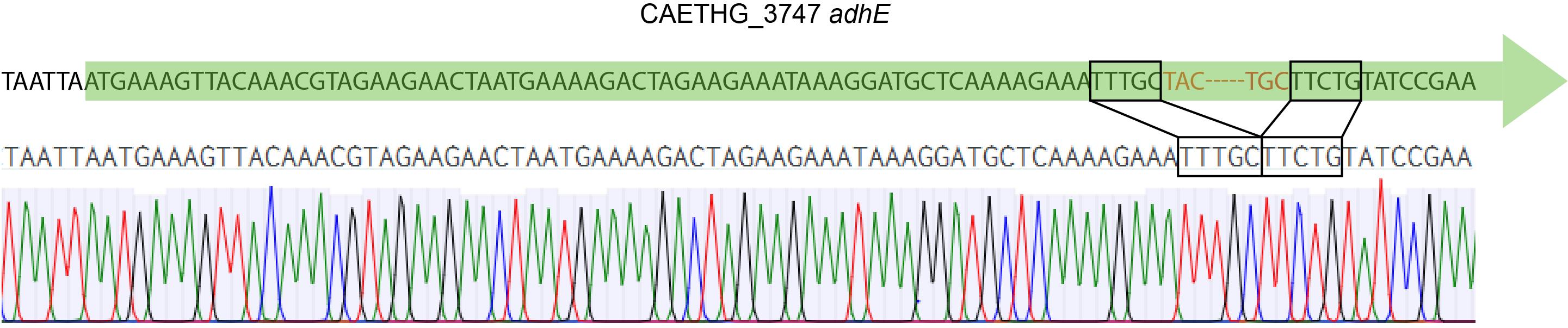


Figure S 2. **Image of Sanger sequencing results showed the nature of the in-frame deletion in the Ald subunit of the adhE gene (CAETHG_3747).**


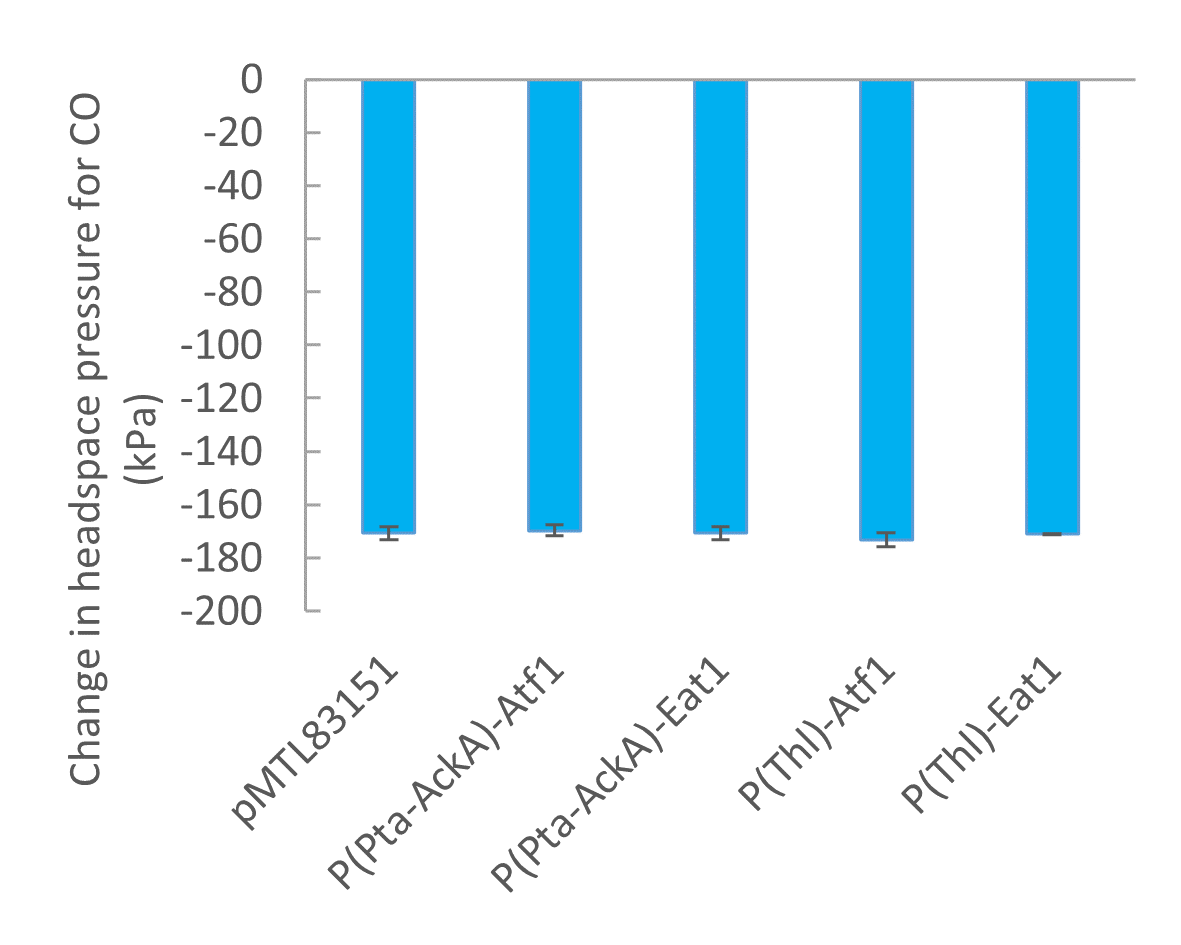


Figure S. 3. **Change in CO headspace pressure for C. autoethanogenum strains carrying plasmids for AAT expression on CO as main carbon source**. Data is represented as the average ± standard deviation of biological triplicates.


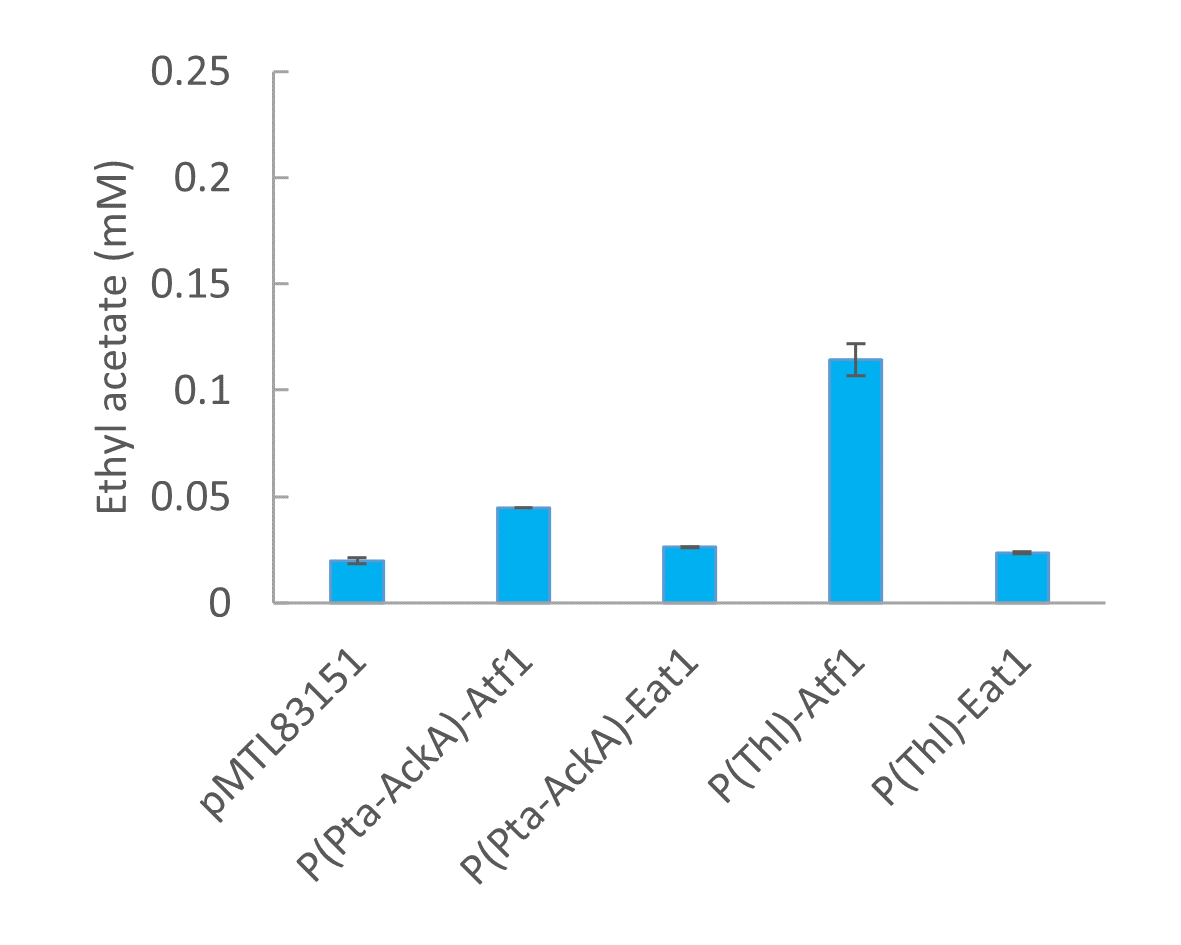


Figure S 4**. Screening of ethyl acetate production by C. autoethanogenum strains carrying plasmids for AAT expression on 40 mM fructose**. Data is represented as the average ± standard deviation of biological triplicates.


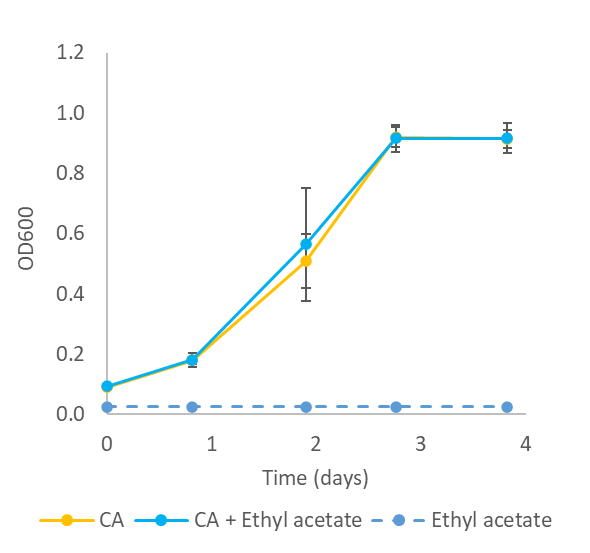


Figure S 5. **Investigating growth of C. autoethanogenum on CO and ethyl acetate**. OD600 over time for C. autoethanogenum (CA) grown on CO with or without addition of approximately 4 mM ethyl acetate, including a negative control without presence of C. autoethanogenum. Data is represented as the average ± standard deviation of biological triplicates.


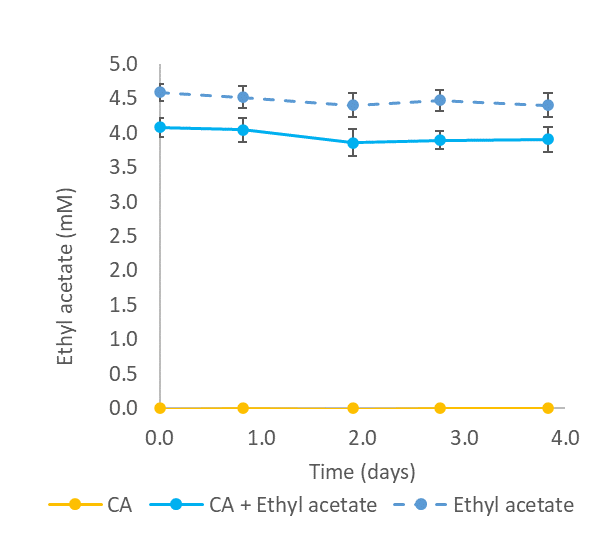


Figure S 6. **Investigating ethyl acetate degradation by C. autoethanogenum grown on CO.** Ethyl acetate degradation over time for ethyl acetate with or without presence of C. autoethanogenum (CA) during growth on CO including a control with only C. autoethanogenum and no ethyl acetate. Data is represented as the average ± standard deviation of biological triplicates.


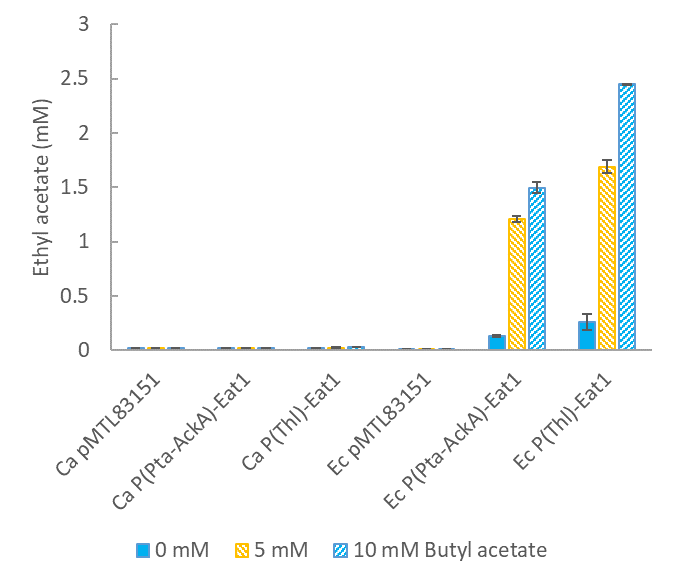


Figure S 7. **Eat1 in vivo alcoholysis assay for C. autoethanogenum and E. coli**. C. autoethanogenum (Ca) was grown on CO and E. coli (Ec) grown in LB expressing Eat1 with either 0 mM, 5 mM, or 10 mM butyl acetate supplementation. Data is represented as the average ± standard deviation of biological triplicates.


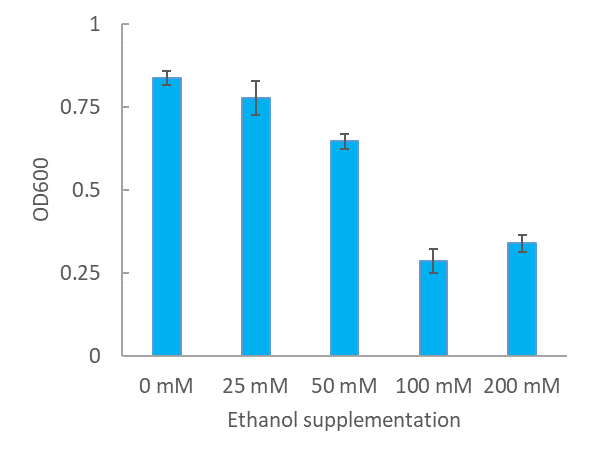


Figure S 8. **Investigating the effects of ethanol supplementation on growth of C. autoethanogenum [P_Thl_-Atf1] grown on CO**. OD600 for C. autoethanogenum [P_Thl_-Atf1] incubated for 7 days on CO with varying concentrations of ethanol supplementation. Data is represented as the average ± standard deviation of biological triplicates.


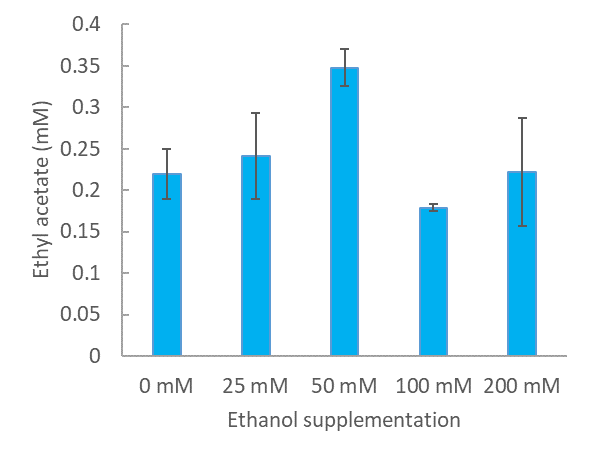


Figure S 9**. Investigating the effects of ethanol supplementation on ethyl acetate production by C. autoethanogenum [P_Thl_-Atf1] grown on CO**. Final ethyl acetate production for C. autoethanogenum [P_Thl_-Atf1] grown on CO with varying concentrations of ethanol supplementation. Data is represented as the average ± standard deviation of biological triplicates.
